# A nationwide neurosurgical inter-disciplinary service for cancer-related refractory pain

**DOI:** 10.1186/s12904-024-01501-8

**Published:** 2024-07-20

**Authors:** Morsi khashan, Ido Strauss, Yehonathan Hochberg, Silviu Brill, Rotem Tellem, Haggai Sharon, Uri Hochberg

**Affiliations:** 1 Sackler School of Medicine, Tel Aviv University, Tel Aviv, Israel; 2 Department of Neurosurgery, Spine Unit, Tel Aviv Sourasky Medical Center, Tel Aviv, Israel; 3 Institute of Pain Medicine, Division of Anesthesiology, Department of Anesthesiology and Critical Care Medicine, Tel Aviv Sourasky Medical Center, Tel Aviv, Israel; 4 The Palliative Care Service, Tel Aviv Sourasky Medical Center, 6 Weizmann Street, Tel Aviv, Israel

**Keywords:** Cancer pain, Interdisciplinary, Ablative procedures, Palliative care, Refractory pain

## Abstract

**Purpose:**

Neurosurgical ablative procedures, such as cordotomy and cingulotomy, are often considered irreversible and destructive but can provide an effective and individualized solution for cancer-related refractory pain, when all other approaches have been unsuccessful. This paper provides an in-depth exploration of a novel approach to managing refractory cancer pain. It involves an interdisciplinary team led by a neurosurgeon at a renowned national referral center.

**Methods:**

a retrospective analysis of the medical records of all sequential patients who underwent their initial evaluation at our interdisciplinary refractory cancer pain clinic from February 2017 to January 2023.

**Results:**

A total of 207 patients were examined in the clinic for a first visit during the study period. All patients were referred to the clinic due to severe pain that was deemed refractory by the referring physician. The mean age was 61 ± 12.3 years, with no significant sex difference (*P* = 0.58). The mean ECOG Performance Status score was 2.35. Conservative measures had not yet been exhausted in 28 patients (14%) and 9 patients were well controlled (4%). Neurosurgical ablative procedures were recommended for 151 (73%) of the patients. Sixty-six patients (32%) eventually underwent the procedure. 91 patients (44%) received a negative recommendation for surgery. Thirty-five patients (17%) were referred for further invasive procedures at the pain clinic.

**Conclusion:**

An Interdisciplinary cooperation between palliative care specialists, pain specialists, and neurosurgeons ensures optimal patient selection and provides safe and effective neurosurgery for the treatment of refractory cancer-related pain.

## Introduction

In recent years, significant progress has been made in the treatment of cancer patients, with new biological and immunological therapies and improved supportive care leading to long-term survival, even for those in advanced stages. However, pain management remains a major challenge for patients with advanced metastatic disease and their care teams.

Cancer-related pain is prevalent among patients, especially those with advanced metastatic disease. While the WHO guidelines provide effective pain relief for most patients, 10–20% of patients experience refractory pain or intolerable side effects from medication [1, 2]. Refractory cancer pain is a challenging situation that lacks established treatment guidelines [3], and which is commonly managed by palliative services with high dose analgesics and their considerable adverse effects. Evidence suggests that integrating palliative care into standard cancer care can improve outcomes for patients and caregivers and should be part of a comprehensive cancer care plan [4]. Interventional procedures, such as epidural spinal injection and nerve blocks, are commonly employed by pain specialists, anesthesiologists and neurosurgeons to manage cancer-related pain. More complex neuromodulatory.

techniques like spinal cord stimulation (SCS) and intra-thecal pumps may also be utilized [5–7]. Neurosurgical ablative procedures, such as percutaneous cordotomy, stereotactic cingulotomy, and stereotactic mesencephalotomy, are often considered destructive and irreversible since the implementation of neuromodulatory techniques fell out of favor [8, 9]. However, they can offer an effective and individualized solution for refractory cancer pain when other approaches have failed. Cordotomy and mesencephalotomy specifically targets the spinothalamic tract to interrupt pain perception [10], while cingulotomy ablates neural pathways in the cingulate gyrus, a brain region responsible for emotional pain perception, to achieve pain reduction through central pain modulation [11].

In 2015 we established an interdisciplinary clinic for refractory cancer-related pain comprising a team of a palliative care specialist, nurse practitioner, neurosurgeon, and a pain specialist. The clinic evaluates and selects patients for neurosurgical ablative pain procedures and has become a referral center for patients with advanced metastatic disease requiring immediate pain relief. Recently, the safety and efficacy of these procedures for appropriately selected cancer patients were demonstrated [12]. However, the limited adoption of these procedures may be attributed to the insufficient knowledge and awareness among physicians regarding the procedures, their expected outcomes, and the criteria for patient eligibility.

This paper outlines the framework and collaboration with a neurosurgeon aimed at relieving refractory cancer-related pain.

## Methods

### Study design and population

We retrospectively reviewed the medical records of all consecutive patients who were evaluated for the first time at our interdisciplinary intractable cancer pain clinic between February 2017 and January 2023. The data collected refers to the patients’ first visit to the clinic. The study received the approval of the Research Ethics Board of our institution (The Tel-Aviv Souraski Medical Centre. Research, Development, and innovation division, Helsinki committee, approval number: IR50354-17).

Demographic variables, and oncological disease variables were collected as well as data regarding the interventional procedures. To evaluate the functional status of the patients we collected data regarding patients’ ambulation and basic activity of daily living (BADL). The Eastern Cooperative Oncology Group (ECOG) performance status scoring system was utilized to evaluate the patient’s functional status. This score is routinely used in clinical practice. The scores range from 0 to 5, with 0 indicating asymptomatic patients, 4 indicating a bedridden patient and 5 indicating mortality.

We further divided the patients into two groups. patients who underwent surgery were included in “Surgery” group and patients who were treated conservatively were included in “No surgery” group. We then compared these groups in terms of demographic variables, the presence of metastatic disease and active treatment.

All patients were assessed by an interdisciplinary team comprising of a palliative care specialist, nurse practitioner, neurosurgeon, and a pain specialist.

### Statistical analysis

The statistical analysis was performed using SPSS version 28 (IBM Corp., Armonk, NY). Significant differences between the groups were determined using independent samples t-test, the X2 test, and the Fisher exact test, to evaluate categorical variables’ independence. A p-value < 0.05 was considered statistically significant.

### Large language models

We used ChatGPT (https://openai.com/blog/chatgpt) lightly solely for improved readability purposes. The usage was carried out with human oversight and control, with careful review and editing of the content and result.

## Results

### Demographics, disease

A total of 207 patients were examined in the clinic for a first visit during the study period. ninety-nine were females (Table 1a) The mean age was 61 ± 12.3 years, with no significant difference between sexes (61.8 ± 12.4 Vs. 60.2 ± 12.2 years, respectively, *P* = 0.58) (Table 1b). The mean age did not differ significantly between the patients who underwent a neurosurgical procedure and those who did not (59.9 and 61.5 years, respectively, *P* = 0.21). 94 out the 207 patients in the study (45.4%) were referred from other medical centers across the country, as our medical center is the only one providing these neurosurgeries. Metastatic disease was found in 192 (92%) patients and 137 patients (66%) were during active oncological treatment (systemic or radiation) at the time of the first consultation. The most common malignancy was lung (24%) followed by the genitourinary tract (15%) and gastrointestinal tract (14%). Most patients (93%) suffered from metastatic disease.

**Table 1:** Demographics

Table 1A			Table 1B				
**Total cohort**		207			**Surgery**	**No Surgery**	
**Sex**	Women	99 (48%)	**Total**		65	142	p-value
	Men	108 (52%)	**Age**		59.9 ± 12	61.5 ± 12.5	0.613
**Age**	Total	61 ± 12.30	**Male gender**		32 (49%)	73 (51%)	0.766
	Women	60.2 ± 12.20	**Metastatic disease**	192 (92%)	64 (33%)	128 (67%)	0.508
	Men	61.8 ± 12.40	**Active treatment**	137 (66%)	42 (30%)	95 (69%)	0.574
**Diagnosis**	Sarcoma	23 (11%)					
	Lung	49 (24%)					
	Hematological	6 (3%)					
	UnKnown	6 (3%)					
	Breast	27 (13%)					
	Gastrointestinal	32 (15%)					
	Genitourinary	29 (14%)					
	Head and Neck	7 (3%)					
	Gynecological	8 (4%)					
	Other	20 (10%)					
**Metastatic**		192 (93%)					
**Active treatment**		137 (66%)					

### Functional status

The mean Eastern Cooperative Oncology Group (ECOG) Performance Status score [13] was 2.35 for the entire cohort, and 26 (12%) of the patients had a score of zero or 1 (asymptomatic or symptomatic but fully mobile). Eighty-eight (43%) of the patients needed some assistance in the activities of BADL. Eighty-two patients (40%) were walking independently, 64 patients (31%) were wheelchair or bed bound ***(***Table 2**)**.

**Table 2:** Functional status

Functional status	Total cohort	207
**ECOG**	0- Fully active	5 (2.4%)
	1- Restricted but able to carry out light work	21 (10.1%)
	2- up and about more than 50% of waking hours	95 (46%)
	3- confined to bed/chair > 50% of waking hours	67 (32.3%)
	4- Completely disabled	19 (9.1%)
**BADL**	Independent	85 (41%)
	Assisted	88 (43%)
	Dependent	34 (16%)
**Ambulation**	Independent	82 (39.6%)
	Cane (assisted)	26 (13%)
	Walker (assisted)	35 (17%)
	Wheelchair (non-ambulatory)	60 (29%)
	Bedridden (non-ambulatory)	4 (2%)

### Team assessment and recommendations

All patients were referred to the clinic due to severe pain that was deemed refractory by the referring physician. The interdisciplinary team evaluation concluded that 170 patients (82%) indeed suffered from refractory pain. In 110 patients (53%) the team’s impression was that the pain was refractory to adequate trials of high doses of opiates, while in 60 patients (29%) this was due to drug intolerance. High dose opioids are considered when patients receive more than 90 mg MEDD, which is an acceptable benchmark [14]. The team concluded that conservative measures had not yet been exhausted in 28 patients (14%) and that 9 patients were well controlled (4%). We could not find any correlation between the primary diagnosis and the recommendation for surgery or the actual execution of the surgery.

Neurosurgical ablative procedures for pain control were recommended for 151 (73%) of the patients. Sixty-six patients (32%) eventually underwent the procedure. 50 patients were found to be eligible for intervention but did not undergo surgery as detailed in Table 3. 55 patients (27%) received a negative recommendation for surgery. Thirty patients (14%) were referred for further minimally invasive treatment (including spinal cord stimulation and intra-thecal pump insertion at the pain clinic). Eleven patients were re-evaluated prior to surgery based on varying recommendations of which two patients were referred to pain clinic for nerve block, two received oncological orthopedic surgery, one received radiation and the remaining six patients received additional pharmaceutical treatment (Table 3).

**Table 3:** Team assessment and recommendations

	Total cohort	207
**Assessment**	Lack of response to prior pharmaceutical treatment	110 (53%)
	Drug intolerance	60 (29%)
	Conservative treatment not exhausted	28 (14%)
	Balanced pain control	9 (4%)
**Recommendations**	**Positive recommendation for intervention**	**151 (73%)**
	Underwent surgery	66 (32%)
	Referral to Pain Clinic	35 (17%)
	Recommendation for intervention but not carried out	50 (24%)
	Lost to follow up	31 (15%)
	Refused by patient	13 (6%)
	Died prior to procedure	6 (3%)
	**Negative recommendation for intervention**	**55 (27%)**
	High risk	15 (3%)
	Other measures recc	30 (14%)
	Other surgical evaluation	11 (5%)

## Discussion

Selecting patients for palliative neurosurgical interventions for refractory cancer-related pain can be challenging and require balancing different treatment priorities.

We present our interdisciplinary teamwork model that consists of specialists from 3 different disciplines simultaneously interacting with the patient during the same clinic visit. We demonstrate in this work the collaboration between different specialists, each bringing their point of view to the discussion allows for more comprehensive attitude toward patients with complex oncological disease.

Although all patients were referred to our clinic for evaluation for neurosurgical interventions for pain deemed refractory by the referring physician, in almost 1 in 5 patients (18%) the conclusion was that either pharmaceutical options were not yet exhausted or that the pain was tolerable. This brings up the question of how quickly other physicians may classify patients as “refractory,” emphasizing the significance of earlier referrals and interdisciplinary evaluations. Moreover, this divergence in assessment may also indicate one of the most typical hindrances to effective cancer-related pain management, which is insufficient healthcare provider knowledge regarding pain management [15, 16]. This discrepancy in pain assessment may also be related to patients’ own differing assessments from their physicians, potentially due to unrealistic expectations or catastrophic thinking [17].

The interdisciplinary approach is defined as “a synthesis of two or more disciplines, establishing a new level of disclosure and integration of knowledge” [18]. This approach promotes open communication and collaboration among the healthcare team, enabling better overall care for the patient. At our Tripartite collaboration of the disciplines, the Palliative care physicians provide a holistic approach focusing on managing symptoms and improving quality of life and defining the patients’ goals of care. The invasive pain specialists use a variety of minimally invasive procedures for pain relief and improve function, while neurosurgeons use surgical neuro-ablative techniques to relieve pain.

**Fig. 1:**
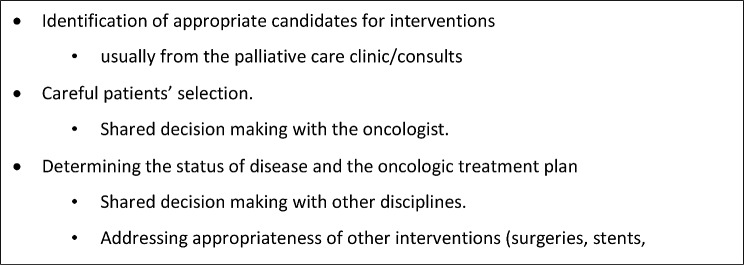
Inter-Disciplinary team’s goals

The service aims to evaluate each patient’s individual case and weigh the potential benefits and risks of different interventional procedures, including neurosurgical ablations, to identify the best candidates who may benefit the most from these procedures. This provides access to these procedures for patients with advanced oncological disease and refractory pain who would otherwise be ineligible due to their complex medical condition, offering an alternative to extreme interventions such as palliative sedation **(**Fig. 1**)**.

As anticipated, the usual patient referred to our specialized clinic had advanced metastatic disease and experienced severe pain due to cancer. Most patients were middle-aged with some degree of functional impairment, yet still able to walk, with an average of less than half their time spent in bed. A significant proportion of patients (66%) were undergoing ongoing oncological treatment, which required collaboration with oncologists and occasionally caused postponement of the surgery decision or implementation, creating unique obstacles in achieving the patients’ care goals.

Interestingly, when examining the different tumor pathologies, Sarcomas, a heterogeneous group of soft tissue malignancies that account for only 1% of the overall malignancies, represented 11% of patients at our clinic. This might be due to our medical center serving as a country-wide referral to these patients, or because of the tendency of these types of tumors to cause severe pain [19]. On the other hand, although breast cancer is the most common malignancy worldwide, it was only the fourth most common among the patients who were presented to our clinic (Tables 1 and 2).

**Fig. 2:**
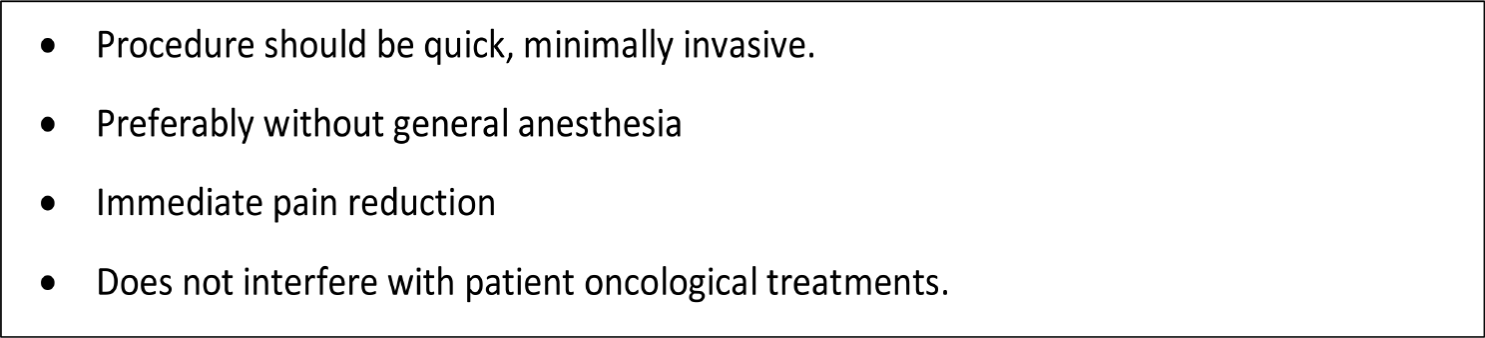
Procedures for terminally ill oncological patients

The initial pain clinic visit involved evaluating the patient’s pain in a holistic manner, based on the concept of “total pain” [20] considering physical, psychological, social, and spiritual factors along with their care plan and goals. Following the assessment and consideration of the patient’s prognosis and disease status, they were either recommended for neurosurgical ablative procedures or offered less invasive alternatives such as minimally invasive or noninvasive treatments. 31% of patients were recommended minimally invasive procedures or were found to have underutilized conservative treatments. 35 patients (17%) were referred for minimally invasive interventions such as nerve blocks, soft tissue injections or neuromodulatory procedures including SCS implants and intra-thecal pumps and didn’t return for follow-up. The remaining 30 patients (14%) received supplementary treatments like cognitive behavioral therapy or physiotherapy. This might suggest the underutilization of pain specialists by the primary physicians (usually a oncologists) due to various possible factors: lack of knowledge regarding the variety and safety of the procedures, reluctance to provide referrals for interventions (despite being palliative in nature) for complex and frail patients and the lengthy waitlists for invasive pain specialists [20].

**Fig. 3:**
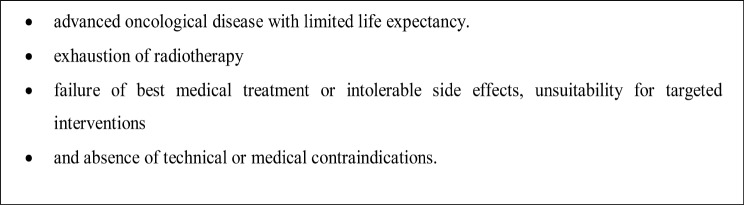
Neurosurgical ablative procedures, selection criteria

While Neurosurgical ablative procedures are effective, they are not without considerable risks. As oncological patients are often frail, with short life expectancy and at considerable risk for medical interventions, patient’s safety is the top priority when considering invasive pain management procedures (Fig. 2). Our interdisciplinary clinic evaluates and selects patients for these procedures (Fig. 3) as not all patients are suitable for neuromodulation techniques due to technical limitations such as spinal pathology that may prevent catheter placement and space constraints for battery/reservoir implantation in cachectic patients. Furthermore, Neurosurgical ablative procedures usually do not necessitate multiple clinic visits.

The two most common neurosurgical ablative procedures provided by our team are cordotomy and cingulotomy. Percutaneous cervical cordotomy (PCC), ablation of the spinothalamic pain pathway at the C1-C2 level, provides immediate unilateral pain relief, can be performed with the assistance of an experienced anaesthesiologist using only remifentanil to minimize sedation, but requires patient cooperation and ability to lie supine for about 1–2 h. An open thoracic cordotomy is an option for patients who cannot undergo other procedures and involves a spine surgery that requires intraoperative electrophysiological neuromonitoring. Open cordotomy was chosen in cases where patients were unwilling to risk upper-limb paresis with PCC, had a fear of needles that prevented them from undergoing an awake PCC, or had a suspected tumour at the entry site at the C1-2 spinal level. Mesencephalotomy may be recommended for patients with pain above the C4 level, those who are unable to lie supine, or when percutaneous cordotomy is not feasible for technical reasons. It is a preferable option to cordotomy in cases of contralateral lung dependence, as cordotomy may cause ipsilateral phrenic nerve damage. Our service has successfully performed mesencephalotomy on three patients, all of whom achieved satisfactory outcomes based on their goals of care.

Diffuse or bilateral pain poses a challenge as treatment options are limited. Bilateral cordotomy is an option but has relatively high morbidity [21]. A preferred alternative is stereotactic cingulotomy. The procedure is performed under sedation and local anesthesia and does not require patient cooperation. Although patients may still experience pain following the procedure, they report improved tolerance and reduced suffering. Over the years, we have found cingulotomy a successful alternative when cordotomy failed. However, it is important to note that recovery is challenging with a two-week period of apathy and mild delirium requiring support from care providers and family members.

Most patients received a comprehensive evaluation only once, and a small number (11) received a reevaluation, in addition to the post-operative follow-up visit in the clinic. Although we typically did not administer pharmaceutical pain management, we recommended adjustments to the current treatment plan to the referring physician if it was deemed beneficial for the patient’s overall pain control. If a patient returns for a second visit and an ablative procedure has not yet been considered, the treatment plan is regularly reviewed and adjusted as necessary. A considerable number of patients were lost to follow-up and did not undergo the recommended ablative neurosurgery, possibly due to fear of the procedure and logistical difficulties associated with traveling from other medical centers. Furthermore, many patients had poor prognoses and experienced medical deterioration that hindered their ability to undergo the surgery.

Holistic decision-making has enabled innovative palliative care for highly complex patients previously considered refractory. The absence of international consensus and standardization in the classification and treatment of refractory cancer-related pain emphasizes the need for interdisciplinary collaboration to optimize patient selection. Therefore, it is essential to implement an integrated pain management and palliative care service to provide these patients with safe and effective interventions that can enhance their quality of life [22]. Many of these patients experienced reduced dependence on opioids, improved functionality, and even resumed cancer treatments that were previously halted due to uncontrolled pain. This aligns with the modern palliative care philosophy, as stated by Dame Cicely Saunders, the founder of the hospice movement:

“ You matter because you are you, and you matter to the end of your life. We will do all we can not only to help you die peacefully, but also to live until you die“ [23].

It’s important to note several limitations of this study, including its retrospective design and the absence of data on other functional parameters and survival time after the visit. Additionally, due to the short follow-up period, the long-term effects of these interventions remain unclear. Another limitation of this study is its small size, which precludes us from performing subgroup analyses to further specify the current indications and various syndromes, such as midline pelvic pain or diffuse bilateral pain.

## Conclusion

Interdisciplinary cooperation between palliative care specialists, pain specialists, and neurosurgeons is crucial to ensure optimal patient selection and provide safe and effective neurosurgery for the treatment of refractory cancer-related pain. Patient selection considerations, patient safety, and personal preferences should be carefully considered in determining the best course of action, ensuring the best possible outcomes for patients.

## Data Availability

The data presented in this study are available on request from the corresponding author.
